# Persistent post‐COVID headache is associated with suppression of scale‐free functional brain dynamics in non‐hospitalized individuals

**DOI:** 10.1002/brb3.3212

**Published:** 2023-10-23

**Authors:** Nathan W. Churchill, Eugenie Roudaia, J. Jean Chen, Asaf Gilboa, Allison Sekuler, Xiang Ji, Fuqiang Gao, Zhongmin Lin, Mario Masellis, Maged Goubran, Jennifer S. Rabin, Benjamin Lam, Ivy Cheng, Robert Fowler, Chris Heyn, Sandra E. Black, Bradley J. MacIntosh, Simon J. Graham, Tom A. Schweizer

**Affiliations:** ^1^ Neuroscience Research Program, St. Michael's Hospital Toronto Ontario Canada; ^2^ Keenan Research Centre for Biomedical Science, St. Michael's Hospital Toronto Ontario Canada; ^3^ Physics Department Toronto Metropolitan University Toronto Ontario Canada; ^4^ Rotman Research Institute Baycrest Academy for Research and Education Toronto Ontario Canada; ^5^ Department of Medical Biophysics University of Toronto Toronto Ontario Canada; ^6^ Institute of Biomedical Engineering University of Toronto Toronto Ontario Canada; ^7^ Department of Psychology University of Toronto Toronto Ontario Canada; ^8^ Department of Psychology, Neuroscience & Behaviour McMaster University Hamilton Ontario Canada; ^9^ LC Campbell Cognitive Neurology Research Group, Sunnybrook Health Sciences Centre Toronto Ontario Canada; ^10^ Hurvitz Brain Sciences Program Sunnybrook Research Institute Toronto Ontario Canada; ^11^ Physical Sciences Platform Sunnybrook Research Institute Toronto Ontario Canada; ^12^ Division of Neurology, Department of Medicine, Sunnybrook Health Sciences Centre University of Toronto Toronto Ontario Canada; ^13^ Harquail Centre for Neuromodulation Sunnybrook Research Institute Toronto Ontario Canada; ^14^ Rehabilitation Sciences Institute University of Toronto Toronto Ontario Canada; ^15^ Evaluative Clinical Sciences Sunnybrook Research Institute Toronto Ontario Canada; ^16^ Integrated Community Program Sunnybrook Research Institute Toronto Ontario Canada; ^17^ Department of Medicine University of Toronto Toronto Ontario Canada; ^18^ Emergency & Critical Care Research Program Sunnybrook Research Institute Toronto Ontario Canada; ^19^ Department of Medical Imaging University of Toronto Toronto Ontario Canada; ^20^ Computational Radiology & Artificial Intelligence Unit, Division of Radiology and Nuclear Medicine Oslo University Hospital Oslo Norway; ^21^ Faculty of Medicine (Neurosurgery) University of Toronto Toronto Ontario Canada

**Keywords:** brain function, COVID‐19, fMRI, headache, scale‐free

## Abstract

**Introduction:**

Post‐acute coronavirus disease 2019 (COVID‐19) syndrome (PACS) is a growing concern, with headache being a particularly debilitating symptom with high prevalence. The long‐term effects of COVID‐19 and post‐COVID headache on brain function remain poorly understood, particularly among non‐hospitalized individuals. This study focused on the power‐law scaling behavior of functional brain dynamics, indexed by the Hurst exponent (*H*). This measure is suppressed during physiological and psychological distress and was thus hypothesized to be reduced in individuals with post‐COVID syndrome, with greatest reductions among those with persistent headache.

**Methods:**

Resting‐state blood oxygenation level‐dependent (BOLD) functional magnetic resonance imaging data were collected for 57 individuals who had COVID‐19 (32 with no headache, 14 with ongoing headache, 11 recovered) and 17 controls who had cold and flu‐like symptoms but  tested negative for COVID‐19. Individuals were assessed an average of 4–5 months after COVID testing, in a cross‐sectional, observational study design.

**Results:**

No significant differences in *H* values were found between non‐headache COVID‐19 and control groups., while those with ongoing headache had significantly reduced *H* values, and those who had recovered from headache had elevated *H* values, relative to non‐headache groups. Effects were greatest in temporal, sensorimotor, and insular brain regions. Reduced *H* in these regions was also associated with decreased BOLD activity and local functional connectivity.

**Conclusions:**

These findings provide new insights into the neurophysiological mechanisms that underlie persistent post‐COVID headache, with reduced BOLD scaling as a potential biomarker that is specific to this debilitating condition.

## Graphical abstract

Post‐acute COVID syndrome is often associated with debilitating headache, but the neural mechanisms are understudied. In this study, the resting brain activity of individuals with post‐COVID headache showed suppression of scaling properties relative to COVID and non‐COVID groups without headache, providing novel insights into this highly prevalent disorder.

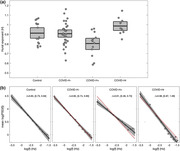

## 1 INTRODUCTION

The coronavirus disease 2019 (COVID‐19) pandemic has created an ongoing public health crisis, with the disease potentially having long‐lasting health effects for infected individuals. A particular growing concern is post‐acute COVID‐19 syndrome (PACS), typically defined as COVID‐related symptoms and neurological disturbances that persist over 12 weeks post infection (Shah et al., 2021; World Health Organization, 2021). This disorder is highly prevalent among survivors of COVID‐19, with persistent symptoms reported in 30% or more of individuals, across a range of cohorts (Nalbandian et al., 2021). Although individuals with PACS report a diverse array of symptoms, recent studies have singled out headache as particularly noteworthy, given its high prevalence (Fernández‐de‐las‐Peñas et al., 2021) and its debilitating impact on quality of life (Martelletti et al., 2021; Tana et al., 2022). It has been hypothesized that post‐COVID headache is triggered by inflammation‐mediated activation of the trigeminovascular system among individuals with pre‐existing headache and/or a genetic predisposition to migraine (Straburzyński et al., 2023; Tana et al., 2022), although COVID‐related neural injury may also play a role (Galea et al., 2022). At present, we have an incomplete understanding of the etiology of headache in PACS and its effects on neural function, making this a critical area of ongoing research.

Although understudied in the context of COVID‐19, there is an extensive body of neuroimaging literature examining headache in other conditions, such as migraine, cluster headache, tension‐type headache, and medication overuse headache (e.g., see Chen et al., 2017; Ferraro et al., 2012; Tu et al., 2020; Wang et al., 2014). These studies have frequently reported altered brain function within subcortical and cortical regions implicated in pain processing and modulation (Chong et al., 2019; May, 2006), including the amygdala, thalamus, basal ganglia, anterior insula, anterior cingulate, sensorimotor cortex, prefrontal cortex, and posterior parietal cortex. Much of the literature also shows changes in distributed functional networks that are not specific to headache, such as the default‐mode, salience, and executive control networks (Chong et al., 2019; Maleki & Gollub, 2016), emphasizing that this condition tends to have a global impact on brain functioning. By contrast, previous functional neuroimaging studies of COVID‐19 have identified alterations in brain activity associated with persistent symptoms, including reduced neurometabolism in subcortical regions (Guedj et al., 2021; Sollini et al., 2021) and patterns of altered inter‐regional communication related to olfactory dysfunction (Esposito et al., 2022) and to neuropsychiatric symptoms (Cattarinussi et al., 2022). At present, however, the effects of persistent post‐COVID headache on brain function remain understudied. One promising approach for assessing these effects is through the dynamics of resting brain activity.

In a healthy brain, resting neural activity exhibits scale‐free behavior, in which fluctuations occur across a wide range of timescales, with no scale having a dominant role. The fluctuations are thus “self‐similar” so that if a segment of a time series is taken and magnified, it statistically resembles the whole. Stated more precisely, a time series of brain activity x(t) exhibits self‐similarity if, for any positive scalar value *a*, it is identically distributed with a dilated and scaled version of itself $x\left(t\right)\;∼\;a^{-H}x\left(at\right)$. Scaling behavior is then fully described by the Hurst exponent *H*, with higher values indicating more scale‐free signal. In signal analysis, scaling is usually defined via a power‐law relationship between frequency *f* and the corresponding power spectral density (PSD), expressed as $PSD\left(f\right)∼{\left|f\right|}^{-\beta }$. The scaling exponent β can then be mapped to the Hurst exponent via the relationship H=(β+1)/2 under a fractional Gaussian noise model (Eke et al., 2000). Other scaling estimators have also been developed, including time‐domain methods (DFA; Peng et al., 1994, 1995) and wavelet‐based techniques (Abry & Veitch, 1998; Abry et al., 1995). In addition, more advanced techniques have been used to describe the spectrum of time‐varying scaling exponents *h* instead of a single exponent *H* (Lashermes et al., 2005; Wendt & Abry, 2007; Wendt et al., 2007). These techniques have been applied to functional neuroimaging data obtained from both healthy and patient cohorts (Ciuciu et al., 2012; He, 2011, 2014; Van de Ville et al., 2010; Wink et al., 2008). Studies have found that scaling behavior is often suppressed (i.e., the exponent *H* is decreased) under conditions of increasing physiological and psychological demand, for example, during challenging and/or unfamiliar tasks (Barnes et al., 2009; Churchill et al., 2016; Ciuciu et al., 2012), with increased age (Churchill et al., 2016), under psychological distress (Churchill et al., 2015; Tolkunov et al., 2010), and following mild traumatic brain injury (Churchill et al., 2020). Such findings suggest that *H* may similarly be sensitive to PACS, in terms of the mental burden of post‐COVID symptoms (Peghin et al., 2021) and any underlying neural injury. This is particularly relevant for persistent headache, which may cause significant psychological distress (Kristoffersen et al., 2018) and tends to interfere with normal cognitive function (Moore et al., 2013; Vuralli et al., 2018).

The present cross‐sectional observational study examined this question using resting‐state blood‐oxygenation‐level dependent functional magnetic resonance imaging (BOLD fMRI) data collected as part of the Toronto‐based NeuroCOVID‐19 study (MacIntosh et al., 2021). The study compared whole‐brain functional dynamics of self‐isolating individuals who tested positive for severe acute respiratory syndrome coronavirus 2 (SARS‐CoV‐2) and subsequently experienced persistent lingering symptoms, relative to controls who had cold or flulike symptoms but tested negative for SARS‐CoV‐2, with both groups imaged an average of 4–5 months after COVID testing. Analysis was conducted after dividing the COVID‐19 group into those who had no headache symptoms (COVID‐H‐), those who had ongoing headache symptoms (COVID‐H+), and those whose headache symptoms were resolved at the time of imaging (COVID‐Hr). These groups were compared in terms of the global mean *H* values and regional *H* values, to enable testing of the hypothesis that post‐COVID‐19 condition produces a graded effect on *H*, with COVID‐H‐ showing reductions relative to controls, COVID‐H+ showing further reductions from the COVID‐H‐, and COVID‐Hr showing similar values to COVID‐H‐. The study design also enabled testing of a second, related hypothesis that the brain regions implicated in COVID‐H+ include consensus regions identified in studies of headache disorder (Chong et al., 2019), i.e, the amygdala, thalamus, basal ganglia, anterior insula, anterior cingulate, sensorimotor cortex, prefrontal cortex, and posterior parietal cortex. Finally, supplemental analyses characterized the relationship between alterations in *H* and other commonly used indices of resting brain function, including BOLD activity in the 0.015–0.08 Hz frequency band, along with indices of local and global functional connectivity.

## 2 MATERIAL AND METHODS

### 2.1 Study participants

Study participants with COVID‐19 were recruited through the Department of Emergency Medicine at Sunnybrook Health Sciences Centre, Toronto, Canada; physician referral; and community advertisements, following a positive COVID‐19 diagnosis. The diagnosis was determined according to local provincial public health procedures (Public Health Ontario, 2021) and included nasopharyngeal or oropharyngeal swab with real‐time reverse transcription polymerase chain reaction (PCR) testing, conducted at a provincially approved facility. Participants were assessed a minimum of 14 days post infection and did not travel in this period. The study also recruited controls who previously had symptoms of viral illness but tested negative for COVID‐19 at that time. Participants were eligible for recruitment if they were between 20 and 75 years of age and living independently. Participants were excluded from the study if they previously had a diagnosis of dementia, neurologic disorder including migraine or headache disorder, severe psychiatric illness, traumatic brain injury or ongoing unstable cardiovascular disease, or if they had a contraindication to magnetic resonance imaging (MRI; e.g., ferromagnetic implant). Recruitment and data collection were carried out between May 2020 and December 2021 and was in accordance with the Canadian Tri‐Council Policy Statement 2. Study procedures, which included participant financial compensation in accordance with standard institutional rates, were conducted with full approval by the Sunnybrook Health Sciences Centre ethics board and with participants giving free and written informed consent.

Within the study cohort, eight of 74 participants (11%; one control, seven with COVID‐19) were vaccinated prior to the study‐relevant viral infection; this was due to the study timeline, in which 64 of 74 participants (86%; 16 control, 48 with COVID‐19) were recruited before vaccines were broadly accessible in Ontario, Canada, as part of the provincial “Phase 2” rollout beginning April 2021 (Office of the Premier, 2021). In addition, only one participant in the study (with COVID‐19) had a prior lab‐confirmed COVID‐19 infection, identified 580 days prior to the study‐relevant infection. Initial testing did not find these participants to be significant outliers in terms of demographics, clinical data, or BOLD data, and thus were retained for further analysis.

### 2.2 MRI data

All participants were imaged at Sunnybrook Health Sciences Centre using a 3 Tesla Magnetom Prisma MRI system (Siemens Healthineers). Structural and functional imaging included a T1‐weighted 3‐dimensional magnetization‐prepared rapid gradient‐echo (MPRAGE) scan (sagittal acquisition, 1.0 mm isotropic voxels) and a 2‐dimensional multi‐slice BOLD fMRI scan (3.5 mm isotropic voxels, 30/2130 ms echo time/repetition time, 250 volumes), respectively, as elements of a much lengthier MRI protocol (MacIntosh et al., 2021). The data were processed using a hybrid pipeline that included advanced normalization tools (http://stnava.github.io/ANTs), analysis of functional neuroimages (AFNI; https://afni.nimh.nih.gov), functional MRI of the brain (FMRIB) software library (https://fsl.fmrib.ox.ac.uk/fsl), and custom in‐house software (see Appendix 1 in the Supporting Information for details of acquisition and preprocessing), with the final data in Montreal Neurological Institute coordinate space, including resampling of voxels at 3 mm isotropic resolution. Analysis was conducted within a mask of gray matter voxels and further restricted to exclude the cerebellum, thereby focusing on cortical and subcortical dynamics. Outlier imaging data were identified in terms of both estimated head motion and BOLD signal fluctuations, with two participants (controls) excluded from imaging analysis; further post hoc testing of head motion found no significant confounding effects on the main study analyses (see Appendix 1 in the Supporting Information for details).

### 2.3 Scaling analysis

The overall goal of the analyses was to summarize the dynamics of the spontaneous, arrhythmic fluctuations of resting‐state BOLD time series x(t). One approach involves analyzing signal power at different frequencies *f* using a PSD estimator. However, both the BOLD signal and the underlying neural activity have a broad‐band PSD, in which power declines smoothly with increasing frequency (Fox et al., 2007), hence, no specific set of frequencies or time scales can be singled out for analysis. For this reason, *scaling analysis* is an appealing alternative: instead of examining the absolute BOLD power at a specific frequency band, this approach characterizes the relationships between relative BOLD power at different frequences in terms of power‐law scaling behavior. By convention, x(t) is deemed *scale invariant* if the expression $\mathrm{PSD}\left(f\right)=C{\left|f\right|}^{-\beta }$ holds for a wide range of frequencies, where β>0 and *C* is an arbitrary scaling constant.

This has non‐trivial implications, as the relative spectral content at a given frequency *f* is then entirely dictated by a single scaling exponent β. This parameter is also related to the Hurst exponent by H=(β+1)/2 for a fractional Gaussian noise model (Eke et al., 2000), where *H* is considered an index of temporal dependence. A value of 0.5<H<1.0 indicates positive long‐range autocorrelation in the signal of interest, where a high (or low) value at x(t) tends to be followed by high (or low) values at x(t+1) and subsequent x(t+n). Signals with an *H* value near 1.0 have highly persistent autocorrelations for time lags of n≫1. Conversely, signals with an *H* value near 0.5 show autocorrelations that decay rapidly for n≥1. Values of 0<H<0.5 may also be observed and denote persistent long‐range anticorrelations, where a high (or low) value at x(t) tends to be followed by low (or high) values at x(t+1), with switching behavior extending over time lags of n≫1. In practice, PSD‐based scaling analysis involves plotting log(PSD(f)) values against log(f) values and calculating the linear slope coefficient β from the resulting scatter plot, which is then used to estimate *H*.

For the present study, the PSD was calculated at the voxel level using a Welch estimator (Hanning windowed, with 50% overlap between windows), with band integration over 0.02 Hz intervals to improve estimation stability. The scaling coefficient was then obtained by plotting log(PSD(f)) against log(f) in the range of 0.015 to 0.225 Hz, followed by ordinary least squares estimation of the slope coefficient β. After verifying the appropriateness of the fractional Gaussian noise model (Eke et al., 2000) and linear goodness of fit, the Hurst coefficient *H* was obtained for each voxel. Further testing also affirmed that the scaling relationships identified in this study were not substantially affected by a change in the frequency lower bound (tested: 0.015, 0.02, 0.025, 0.03 Hz), nor the upper bound (tested: 0.225, 0.200, 0.1875, 1750 Hz).

While the main analyses of this study used a PSD‐based estimator of scaling behavior, numerous other scaling estimators exist. Given the ongoing debate surrounding the advantages and drawbacks of different methods in the context of BOLD fMRI, we have also examined BOLD scaling behavior using three other well‐established techniques that have distinct estimation procedures. They include two “monofractal” approaches that describe scaling behavior in terms of a single exponent *H*, along with a “multifractal” approach that summarizes the data in terms of a spectrum of time‐varying scaling exponents. The techniques are detrended fluctuations analysis (DFA; Peng et al., 1994, 1995), wavelet monofractal analysis (WMA; Abry & Veitch, 1998; Abry et al., 1995), and wavelet leader multifractal analysis (WLM; Lashermes et al., 2005; Wendt & Abry, 2007; Wendt et al., 2007). Details of the estimation procedures and the subsequent analysis results are presented in Appendix 2 in the Supporting Information.

### 2.4 Analysis of clinical and demographic data

Participant demographics are listed in Table 1, including age, sex, and years of education; days from symptom onset to imaging, and from PCR test to imaging were also reported. After assessing the approximate normality of demographic data via skewness and kurtosis permutation testing at a threshold of *p*< .05 (5000 resamples), means and standard deviations were reported for measures that did not deviate from normality, and medians with upper and lower quartiles were reported for those that did. All participants completed a questionnaire evaluating symptom status for nine items: fever, cough, sore throat, shortness of breath, fatigue, gastrointestinal issues, problems with smell/taste, headache, and “other.” Symptoms were identified if onset was concurrent with, or subsequent to, the study‐relevant viral infection and PCR test. Participants reported whether each symptom (1) was absent, (2) had occurred but resolved, or (3) was currently ongoing. The present study focused on “headache” as the main symptom of interest. The frequency of ongoing and resolved headache symptoms was reported, along with bootstrapped estimates of the 95% confidence intervals (95%CIs). Note that all bootstrap statistics reported in this study are calculated based on 2000 resampling iterations.

**TABLE 1:** Summary of demographic and clinical data for study participants.

	Control (*N* = 17)	COVID‐H‐(*N* = 32)	COVID‐H+(*N* = 14)	COVID‐Hr (*N* = 11)
Age, mean (standard deviation [SD])	41.5 (13.1) years	40.3 (12.5) years	46.1 (10.1) years	36.2 (10.6) years
Female, total (percent)	10/17 (59%)	21/32 (66%)	11/14 (79%)	6/11 (55%)
Education, mean (SD)	16.8 (2.9)	16.3 (2.0)	17.1 (3.8)	16.5 (1.4)
Days (onset to scan)	180 [138, 218]	151 [92, 198]	112 [80, 155]	126 [110, 189]
Days (test to scan)	141 [59, 221]	120 [74, 193]	112 [67, 147]	124 [109, 183]

*Note*: The mean and standard deviation (SD) are reported for normally distributed data, while the median is reported with lower and upper distribution quartiles [Q1, Q3] for non‐normal data.

Abbreviations: COVID, H‐, coronavirus disease 2019 (COVID‐19) groups without headache; COVID, H+, COVID‐19 groups with ongoing headache; COVID, Hr, COVID‐19 groups with resolved headache.

A series of two‐sample analyses then tested for group differences in demographic variables. The mean differences between groups were calculated, along with bootstrapped standard errors, bootstrap ratios (BSRs; z‐scored statistics of effect, based on the ratio of mean/SE) and two‐tailed percentile *p*‐values; notable differences were identified at an uncorrected threshold of *p* < .05. For the COVID‐19 group, headache was also compared to other non‐headache symptoms. The frequencies of other ongoing symptoms was reported, along with their bootstrapped 95%CIs, and frequencies were compared to headache with reporting of the mean differences, bootstrapped 95%CIs and two‐tailed *p*‐values. Correlations were also calculated between ongoing headache and the presence of other ongoing symptoms, with reporting of Spearman correlation coefficients, bootstrapped 95%CIs and two‐tailed *p*‐values.

### 2.5 Global effects of COVID‐19 on BOLD scaling

For each participant, the global Hurst exponent *H*_glob_ was calculated as the average overall gray matter voxels. To assess for the effects of COVID‐19 and post‐COVID headache on *H*_glob_, a general linear model (GLM) was fitted on the data from all groups (control, COVID‐H‐, COVID‐H+, COVID‐Hr) with three binary regressors denoting membership in the different COVID‐19 subgroups; the GLM also adjusted for effects of age and sex. Coefficients of effect were obtained for each group, along with their bootstrapped distributions. Contrasts were then obtained between all group pairs, with bootstrapped 95%CIs, BSRs, and ‐tailed *p*‐values. Groups with significantly different *H*_glob_ values were identified after adjusting for multiple comparisons at a false discovery rate (FDR) of 0.05. Sensitivity analyses were also conducted, examining the robustness of analysis results under different GLM modeling choices, including the effects of incorporating supplemental demographic and clinical variables; the results are summarized in Appendix 3 in the Supporting Information.

### 2.6 Regional effects of COVID‐19 on BOLD scaling

Subsequent analyses sought to localize the regional effects of COVID‐19 and post‐COVID headache on *H* values, for the group contrasts yielding significant effects on global scaling estimator $H_{\mathrm{glob}}$ . For each voxel in the brain, a GLM was fitted on local *H* values in the same manner as the previous section, obtaining coefficient contrasts, along with bootstrapped 95%CIs, BSRs, and two‐tailed *p*‐values. Then, for each paired group contrast, significant voxels were identified by thresholding at an uncorrected *p*‐value threshold of .005, followed by cluster‐size thresholding at *p* = .05, using the AFNI *3dFWHMx* program to estimate spatial smoothness and the AFNI *3dClustSim* program to estimate the corresponding minimum cluster‐size threshold. For significant brain voxels, the regional effect sizes were then reported in terms of the BSR values and cluster summary reports were also generated.

### 2.7 Comparison with alternative BOLD measures

To provide further context for the observed group differences in scaling behavior, more conventional measures of resting‐state BOLD signal were also examined including the amplitude of low‐frequency fluctuations (ALFF), which computes total BOLD power summed over the range of 0.015–0.08 Hz, and its normalized equivalent, the fractional ALFF (fALFF), which normalizes this power sum by the total spectral power. Both metrics were calculated using the same PSD method as in the scaling analyses. Measures of BOLD functional connectivity were also obtained, including the local connectivity (Lconn), obtained by calculating the pairwise Pearson correlation between all voxel pairs within a region of interest (ROI) and taking the average value, and the global connectivity (Gconn), obtained by taking the mean BOLD time series in an ROI and calculating the mean absolute connectivity with all other voxels in the mask of cerebral grey matter.

These measures were calculated for a 27‐voxel cubic ROI placed on the center of mass for each cluster identified as having significant group differences in scaling effects in the section above, with subsequent averaging over all clusters to obtain a single set of (ALFF, fALFF, Lconn, Gconn) values per participant. Afterward, GLM analyses were conducted to evaluate group differences for each of the BOLD measures as in the previous sections, obtaining coefficient contrasts, along with bootstrapped 95%CIs, BSRs, and two‐tailed *p*‐values.

## 3 RESULTS

### 3.1 Demographic and clinical data

At the time of analysis, 57 participants with COVID‐19 and 17 controls had been recruited and scanned, with T1‐weighted and fMRI data available. Examining symptoms, ongoing post‐COVID headache was reported in 25% (95%CI: [14, 38]%) of the COVID‐19 group and resolved headache reported in 19% (95%CI: [11, 30]%). For the control group, none of the participants had ongoing headache, and only 6% (95%CI: [0, 17]%) had resolved headache. Hence, in the sample under study, the presence of ongoing headache identified individuals with previous COVID‐19 infection among those with persistent complaints, with perfect specificity (100%) but poor sensitivity (25%). Combining ongoing and resolved symptoms slightly reduced specificity (94%) with a modest increase in sensitivity (44%). The demographics are shown in Table 1 for the control and COVID‐19 groups, including those without headache (COVID‐H‐), ongoing headache (COVID‐H+), and resolved headache (COVID‐Hr).

Comparing group demographics (Table 1), the COVID‐H+ group had elevated age relative to the COVID‐Hr group (mean difference and 95%CI: 9.9 years, [2.2, 17.3] years, BSR = 2.47, *p* = .018), but other between‐group age differences were more limited (all |BSR| ≤ 1.71, *p* ≥ .095). There were also no substantial differences between groups in sex (all |BSR| ≤ 1.28, *p* ≥ .218), years of education (all |BSR| ≤ 0.69, *p* ≥ .482), days from symptom onset to imaging (all |BSR| ≤ 2.11, *p* ≥ .089), or days from PCR test to imaging (all |BSR| ≤ 0.79, *p* ≥ .406). Comparing with other self‐reported symptoms (Table 2), headache had a greater ongoing prevalence than fever, sore throat, and gastrointestinal issues but was comparable to other symptoms including cough, shortness of breath, fatigue, and smell/taste issues. Correlating the latter symptoms with headache, only fatigue showed associations at *p* < .05, with all other symptoms showing positive but relatively weak associations. For a summary of “other” symptoms reported by each participant subgroup, see Appendix 4 in the Supporting Information. Although there was substantial reporting heterogeneity between individuals, the COVID‐H+ group tended to report higher frequencies of cognitive issues and body pain/ache relative to other subgroups.

**TABLE 2:** Summary of non‐headache symptom data for study participants.

Symptom	Prevalence	Difference in prevalence from headache	Correlation of prevalence with headache
Fever	0 [0, 0]%	−25 [−14, −36]% (*p* < .001)	–
Cough	14 [5, 23]%	−11 [−25, 4]% (*p* = .113)	0.24 [−0.07, 0.53] (*p* = .124)
Sore throat	4 [0, 9]%	−21 [−33, −9]% (*p* < .001)	–
Shortness of breath	25 [14, 37]%	0 [−16, 17]% (*p* = .877)	0.24 [−0.03, 0.52] (*p* = .082)
Excess fatigue	32 [19, 44]%	7 [−9, 23]% (*p* = .321)	0.40 [0.14, 0.66] (*p* = .003)
Gastrointestinal issues	9 [2, 16]%	−16 [−30, −2]% (*p* = .014)	–
Smell/taste issues	21 [11, 32]%	−4 [−19, 12]% (*p* = .610)	0.11 [−0.16, 0.40] (*p* = .456)

*Note*: For each symptom, prevalence is given as the percentage of the overall coronavirus disease 2019 (COVID‐19) group (*N* = 57) that report ongoing issues, along with a bootstrapped 95% confidence interval (95%CI). The mean difference in prevalence relative to headache is also computed, with a 95%CI and percentile‐based p‐value. For symptoms with prevalence ≥ 10% (and thus stable bootstrap intervals), Spearman correlations with ongoing headache are computed, with bootstrapped 95%CIs and *p*‐values.

### 3.2 Global effects on scale‐free dynamics

**FIGURE 1:**
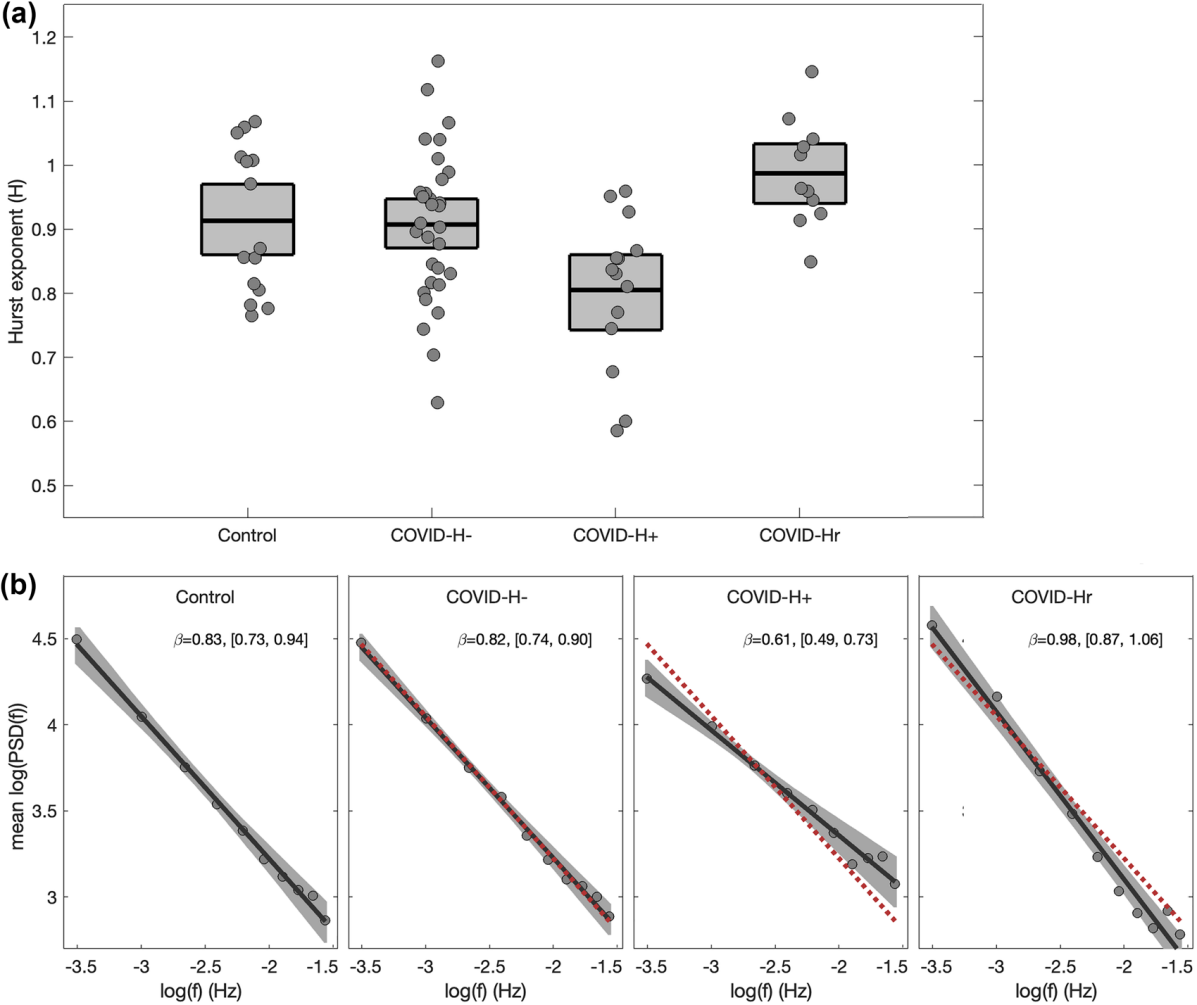
Effects of coronavirus disease 2019 (COVID‐19) and post‐COVID headache on the global Hurst exponent (*H*_glob_). (a) The *H*_glob_ values, calculated over all gray matter, are plotted for controls and COVID‐19 groups without headache (COVID‐H‐), with ongoing headache (COVID‐H+), and resolved headache (COVID‐Hr). Boxes denote group means and bootstrapped 95% confidence intervals (95%CIs) of the mean per group. (b) Frequency scaling behavior shown for each group, with a plot of log spectral power against log frequency, averaged over all participants in each group. A line of best fit is obtained (solid black line) with bootstrapped 95%CIs (shaded areas) for each group, and the line for the control group is overlaid on the COVID‐19 groups (dashed red line) for comparison purposes. The slope of the fitted line β is reported in each panel, along with bootstrapped 95%CIs.

Figure 1a plots the distribution of *H*_glob_ values for the different participant subgroups. The COVID‐H‐ group did not differ significantly from controls (mean difference and 95%CI: −0.007, [−0.081, 0.061], BSR = −0.20, *p* = .867), whereas the COVID‐H+ group was significantly reduced relative to both controls (−0.98, [−0.188, −0.016], BSR = −2.32, *p* = .022) and COVID‐H‐ (−0.091, [−0.165, −0.017], BSR = −2.46, *p* = .012) at an FDR of 0.05. Conversely, COVID‐Hr did not differ significantly from controls (−0.064, [−0.007, 0.145], BSR = 1.63, *p* = .082) but was elevated relative to both COVID‐H‐ (0.071, [0.009, 0.140], BSR = 2.15, *p* = .020) and COVID‐H+ (0.162, [0.079, 0.251], BSR = 3.78, *p* < .001). Sensitivity analyses, reported in Appendix 3 in the Supporting Information, further indicate that these findings are robust under a range of different GLM modeling choices.

Overall, the findings of Figure 1a indicate a consistent reduction in *H*_glob_ for COVID‐H+ and elevated *H*_glob_ for COVID‐H‐. Figure 1b plots the average log spectral power against the log of frequency for each cohort, with a line of best fit displayed, along with the slope coefficient β. Consistent with our interpretation of the scaling exponents *H*, the linear slope is very similar between control and COVID‐H‐ groups with comparable β values, whereas COVID‐H+ shows a shallower slope (smaller β value), and COVID‐Hr shows a steeper slope (larger β value) relative to controls. Similar analyses of *H*_glob_ for the four other symptoms with ongoing prevalence greater than 10% also support the specificity of findings for headache. Fatigue showed effects at an uncorrected *p* < .05 threshold only, in the comparison of COVID‐Hr against COVID‐H‐ (−0.127, [−0.220, −0.022], BSR = −2.54, *p* = .022). No other noteworthy effects were found for any of the other group comparisons and/or symptoms, with all |BSR| ≤ 1.72 and *p* ≥ .074.

### 3.3 Regional effects on scale‐free dynamics

**FIGURE 2:**
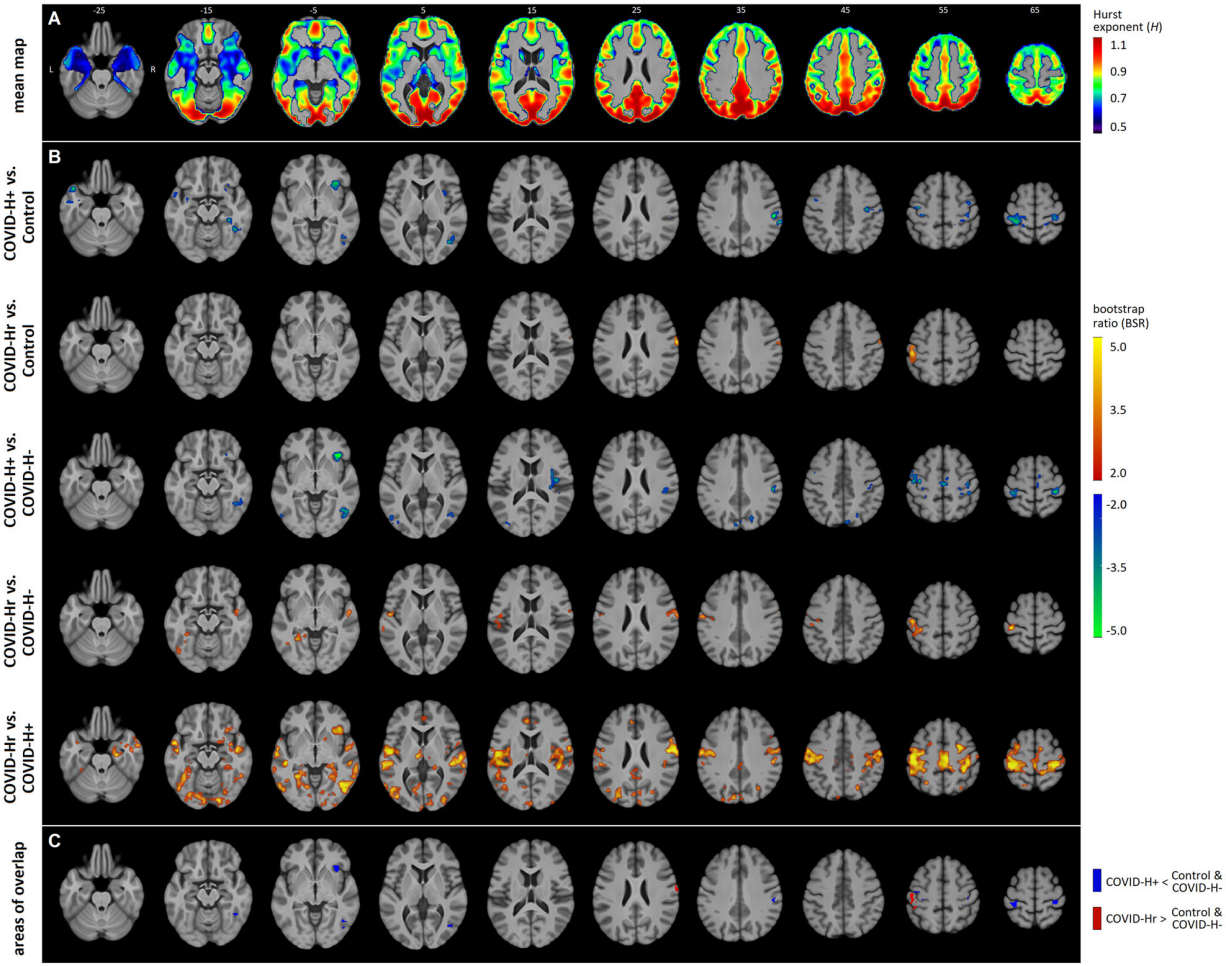
Effects of COVID‐19 and post‐COVID headache on the regional Hurst exponent (*H*). (a) Average map of *H* values, computed over all participants. (b) Areas of significant effect are plotted for each pairwise group contrast, for controls, and COVID‐19 groups without headache (COVID‐H‐), with ongoing headache (COVID‐H+), and resolved headache (COVID‐Hr). Note that the contrast between COVID‐H‐ and control groups was not plotted, as no significant differences were identified. Standardized effect sizes are displayed as z‐distributed bootstrap ratio (BSR) statistics. (c) Consensus areas are depicted where COVID‐H+ and COVID‐Hr groups show significant differences relative to both controls and the COVID‐H‐ group.

Figure 2 displays regional *H* values and between‐group differences. Figure 2a plots the mean *H* map, computed over all participants. Values tended to be highest in the posterior occipital, temporal, and parietal regions, along with the cingulate cortex. Intermediate values are observed frontally, and lower values are observed in subcortical and medial temporal areas. Figure 2b plots brain regions showing significant differences in local *H*. In addition, Figure 2c shows regions where COVID‐H+ and COVID‐Hr groups show significant differences relative to both controls and the COVID‐H‐ group, with clusters summarized in Table 3. Only the comparison of COVID‐H‐ and control groups failed to identify significant differences, which is consistent with the $H_{\mathrm{glob}}$ findings. Comparisons of COVID‐H+ to control and COVID‐H‐ groups consistently show regional decreases in BOLD scaling, with inferior temporal, somatosensory, motor, and insular areas identified in both contrasts. Comparisons of COVID‐Hr to control and COVID‐H‐ groups consistently show regional increases in BOLD scaling, with somatosensory areas identified in both contrasts. Direct comparison of the COVID‐Hr and COVID‐H+ groups yielded the most spatially extensive effects, with previously noted inferior temporal, somatosensory, motor, and insular areas showing more spatially extensive effects, along with regional effects specific to this comparison,  in occipital, thalamic, anterior cingulate, and midcingulate areas.

**TABLE 3:** Significant clusters identified in regional BOLD scaling analysis, shown in Figure 2c.

		Volume (mm^3^)	CoM (mm)	Region (AAL)
COVID‐H+ < Control and COVID‐H‐	1	1917	44, −62, −5	Temporal_Inf_R (ROI 90)
	2	1026	−32, −32, 65	Postcentral_L (ROI 57)
	3	621	32, −32, 62	Postcentral_R (ROI 58)
	4	594	56, −29, 32	SupraMarginal_R (ROI 64)
	5	540	35, 26, −5	Insula_R (ROI 30)
	6	297	−44, −5, 50	Precentral_L (ROI 1)
	7	270	41, −20, 53	Postcentral_R (ROI 58)
	8	135	56, −20, 53	Postcentral_R (ROI 58)
	9	108	−41, −14, 56	Precentral_L (ROI 1)
COVID‐Hr > Control and COVID‐H‐	1	837	−50, −23, 53	Postcentral_L (ROI 57)
	1	702	65, −8, 26	Postcentral_R (ROI 58)
	3	135	53, −11, 32	Postcentral_R (ROI 58)
	4	108	−47, −38, 56	Parietal_Inf_F (ROI 61)

*Note*: Cluster volumes are provided in cubic millimeters (mm^3^), and the cluster center of mass (CoM) is given in Montreal Neurological Institute space coordinates. The brain region is identified for each cluster based on CoM, using the AAL atlas.

Abbreviations: AAL, Automated Anatomical Labeling; BOLD, blood‐oxygenation‐level‐dependent; COVID‐H‐, COVID‐19 groups without headache; COVID‐H+, COVID‐19 groups with ongoing headache; COVID‐Hr, COVID‐19 groups with resolved headache; ROI, region of interest.

Supplemental analyses comparing BOLD scaling effects for different estimators within areas of peak effect are provided in Appendix 2 in the Supporting Information. Comparison of the PSD estimation results to alternative DFA, WMA, and WLM estimators shows comparable directions of effect and similar effect sizes. These results indicate that the observed effects of post‐COVID headache on *H* within consensus brain regions do not depend substantially on the choice of scaling estimator.

### 3.4 Comparison with connectivity and activity measures

**FIGURE 3:**
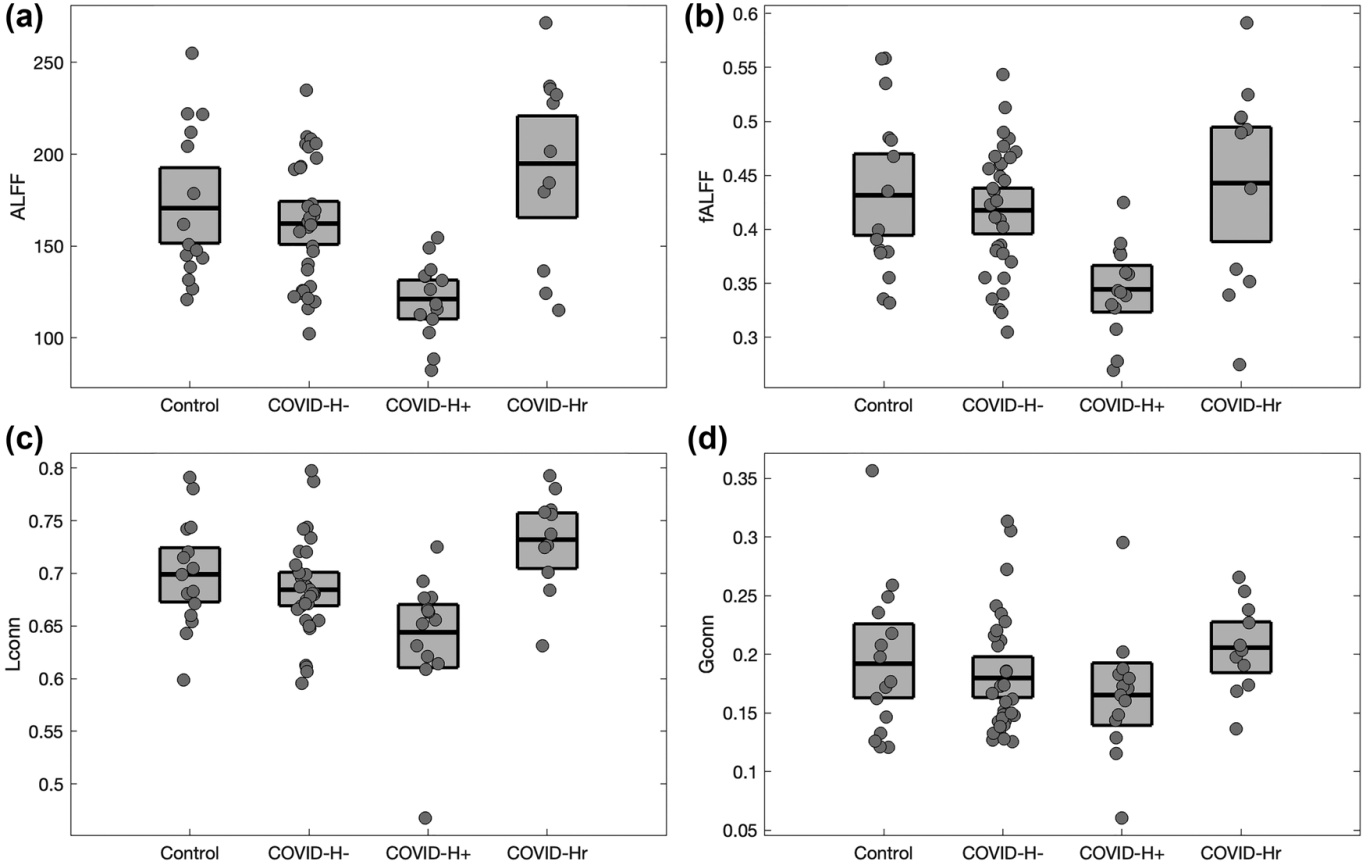
Effects of COVID‐19 and post‐COVID headache on alternative measures of BOLD activity and functional connectivity, for brain regions showing significant effects of ongoing headache (Figure 2c, blue areas). Results are plotted for controls and COVID‐19 groups without headache (COVID‐H‐), with ongoing headache (COVID‐H+), and resolved headache (COVID‐Hr). Results are shown for (a) amplitude of low‐frequency fluctuations (ALFF), (b) fractional ALFF (fALFF), (c) local functional connectivity (Lconn), and (d) global functional connectivity (Gconn). Boxes denote group means and bootstrapped 95%CIs of the mean per group.

For brain regions showing significant BOLD scaling effects of COVID‐H+ relative to controls and COVID‐H‐ (Figure 2c, blue areas), the *H* values exhibited relatively good concordance with ALFF (Spearman *ρ* and 95%CI: 0.89, [0.80, 0.93]), fALFF (0.76, [0.58, 0.88]), Lconn (0.80, [0.69, 0.87]), and Gconn (0.71, [0.56, 0.80]), with Gconn showing the weakest associations. Figure 3 depicts the average values for the different estimators, plotted for significant regions identified in Figure 2c. It can be seen that the effects generalize across estimators, with significantly reduced values in COVID‐H+ relative to both control and COVID‐H‐ groups, seen for ALFF in Figure 3a (−50.10, [−68.30, −33.22], BSR = −5.64, *p* < .001), for fALFF in Figure 3b (−0.092, [−0.127, −0.065], BSR = −5.81, *p* < .001), and for Lconn in Figure 3c (−0.048, [−0.083, −0.017, *p* < .001], BSR = −2.74, *p* = .002) all at an FDR of 0.05, whereas Gconn showed non‐significant decreases in Figure 3d (−0.022, [−0.058, 0.007], BSR = −1.38, *p* = .155). These results indicate that the scaling effects of ongoing headache within consensus brain regions correspond to similar reductions in local measures of resting brain activity and functional connectivity but not Gconn.

**FIGURE 4:**
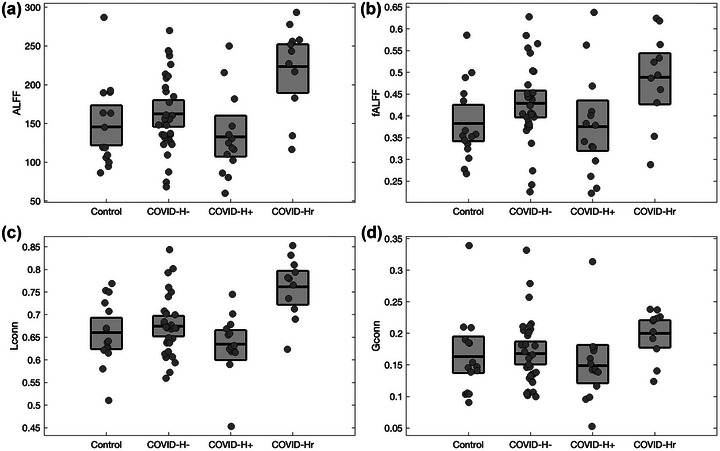
Effects of COVID‐19 and post‐COVID headache on alternative measures of BOLD activity and functional connectivity, for brain regions showing significant effects of resolved headache (Figure 2c, red areas). Results are plotted for controls and COVID‐19 groups without headache (COVID‐H‐), with ongoing headache (COVID‐H+), and resolved headache (COVID‐Hr). Results are shown for (a) amplitude of low‐frequency fluctuations (ALFF), (b) fractional ALFF (fALFF), (c) local functional connectivity (Lconn), and (d) global functional connectivity (Gconn). Boxes denoted group means and bootstrapped 95%CIs of the mean per group.

For brain regions showing significant BOLD scaling effects of COVID‐Hr relative to controls and COVID‐H‐ (Figure 2c, red areas), *H* values exhibited relatively good concordance with ALFF (Spearman *ρ* and 95%CI: 0.92, [0.86, 0.95]), fALFF (0.81, [0.70, 0.89]), Lconn (0.78, [0.67, 0.87]), and Gconn (0.67, [0.49, 0.78]), with Gconn showing the weakest associations. Figure 4 depicts the average values for the different estimators, plotted for significant regions identified in Figure [2c]2d. It can be seen that the effects generalize across estimators, with significantly increased values in COVID‐Hr relative to both control and COVID‐H‐ groups, seen for ALFF in Figure 4a (72.51, [37.06, 107.45], BSR = 4.01, *p* < .001), for fALFF in Figure 4b (0.091, [0.028, 0.154], BSR = 2.81, *p* = .007), for Lconn in Figure 4c (0.095, [0.047, 0.139], BSR = 4.04, *p* < .001), and for Gconn in Figure 4d (0.035, [0.003, 0.065], BSR = 2.09, *p* = .042), all at an FDR of 0.05. These results indicate that the observed scaling effects of recovery from headache within consensus brain regions correspond to similar reductions in local measures of resting brain activity and functional connectivity, along with Gconn.

## 4 DISCUSSION

This study investigated the effects of PACS and persistent post‐COVID headache on functional brain dynamics as indexed by the Hurst scaling exponent *H*. The primary study hypotheses were reduced *H* values in the resting BOLD signal for PACS relative to symptomatic non‐COVID infection, more pronounced decreases in *H* for PACS with ongoing headache symptoms relative to non‐headache PACS, and comparable *H* values in PACS with resolved headache symptoms relative to non‐headache PACS. These expectations are based on a body of literature in which BOLD signal scaling behavior is suppressed under conditions of heightened physiological and psychological burden (Barnes et al., 2009; Churchill et al., 2020, 2016; Ciuciu et al., 2012; He, 2011; Tolkunov et al., 2010). Overall, the hypotheses were partly supported by the analysis results.

The COVID‐19 group without headache did not significantly differ from the control group in terms of global or local *H* values. This suggests that, contrary to study hypotheses, PACS alone does not substantially alter functional brain dynamics relative to symptomatic non‐COVID infection, despite evidence of COVID‐19 targeting brain tissues (Baig et al., 2020; Galea et al., 2022). In terms of the psychological burden, results are also consistent with the similar levels of symptom burden seen in this study's COVID‐19 and control groups as examined in Churchill et al. (2023). However, these findings may be due to the relatively mild sequelae in the current sample of non‐hospitalized individuals with COVID‐19, compared to others who may experience flagrant PACS. Given the high rates of high levels of distress often reported among individuals with PACS (Leviner, 2021), the effects of COVID‐19 on *H* may be more pronounced in other cohorts, including those who were hospitalized while infectious.

Conversely, the COVID‐19 group with ongoing headache had significant global reductions in *H* values relative to both non‐headache COVID‐19 and control groups, indicating altered functional brain dynamics due to post‐COVID headache that were consistent with study hypotheses. The suppression of *H* in BOLD fMRI has been associated with cognitive load during task performance (Barnes et al., 2009; Churchill et al., 2016), with greater effects seen in more challenging conditions relating to task difficulty, task unfamiliarity, and participant age (Churchill et al., 2016). Hence, the present study findings may reflect the greater demands of post‐COVID headache related to, for example, sensory and pain processing. Alternatively, previous studies have identified *H* decreases due to psychological distress (Churchill et al., 2015) and abnormal BOLD scaling for individuals with high trait anxiety (Tolkunov et al., 2010). Given that symptoms of mental distress are common in headache disorders (Kristoffersen et al., 2018), this may also contribute to the effects seen in the present study. Finally, a study of mild traumatic brain injury found decreased *H* during the acute phase of injury (Churchill et al., 2020), along with chronic declines in *H* among the most symptomatic individuals. Similar to PACS, this cohort is likely experiencing a combination of diffuse neural injury and psychological distress relating to post‐concussion symptoms. More broadly, scale‐free dynamics are a property of many biological systems, with links to concepts such as complexity and criticality (Delignières & Marmelat, 2012). Reduced scaling behavior in biological systems generally signifies a loss of system complexity (Goldberger et al., 2002) and thus a more constrained, less adaptive state, which may be vulnerable to further disruption.

Unexpectedly, the COVID‐19 group who had recovered from headache not only had elevated global *H* values relative to COVID‐19 with ongoing headache but also had slightly elevated values relative to the non‐headache COVID‐19 and control groups. It is interesting to note that a similar finding was observed in the previously cited study of mild traumatic brain injury (Churchill et al., 2020), where individuals showed elevated H values at medical clearance. There, it was hypothesized that this represents a relative improvement in mental state, compared to uninjured controls, due to having recently recovered. Given the often debilitating nature of post‐COVID headache (Tana et al., 2022), it is possible that a similar effect is seen here; future studies examining perception of post‐COVID headache and recovery may be informative in interpreting these findings.

Further headache‐related hypotheses posited decreased *H* primarily in areas previously identified across different forms of headache disorder (May, 2006), including the amygdala, anterior cingulate, anterior insula, medial prefrontal, and sensorimotor regions. The present findings again partly support this hypothesis, with alterations in insular and sensorimotor regions reliably identified. The contrast of ongoing versus recovered headache subgroups revealed further effects in the anterior cingulate and amygdala, although the prefrontal cortex was not identified. These areas have been routinely observed in studies of migraine, cluster headaches, and medication overuse headache (Chong et al., 2019). The identified regions are also routinely associated with pain and noxious stimuli, with the somatosensory cortex and amygdala being among the most frequently reported (Iannetti & Mouraux, 2010). Overall, these results support the interpretation that reduced *H* values seen in the COVID‐19 group with headache are at least partly driven by the increased sensory and pain processing demands.

Further analyses using other estimators of the scaling exponent *H* also indicated that the observed effects generalized across all of the tested estimators, including DFA, time‐frequency monofractal methods (WMA) and time‐frequency multifractal methods (WLM). This affirms that the present findings related to post‐COVID headache are not unique to the PSD methodology. This finding is also consistent with prior studies of BOLD scaling behavior, in which the main study conclusions were found to generalize across different estimators of BOLD scaling behavior (Churchill et al., 2015, 2016).

Additional comparison of *H* with other measures indicated that areas of significantly reduced (or increased) BOLD signal scaling associated with post‐COVID headache had similar reductions (or increases) in local BOLD activity and functional connectivity. This is consistent with prior studies showing correlations of *H* with indices of BOLD variability and functional connectivity strength in healthy adults (Churchill et al., 2016; He, 2011). The present results provide further evidence that a change in scaling dynamics of the brain is reflected in other aspects of brain function. For example, the ALFF and fALFF indices reflect the “dynamic range” of the neurovascular response (Yang et al., 2007; Zou et al., 2008), whereas Lconn reflects the local homogeneity of BOLD response and is related to segregative brain function (Zang et al., 2004). Interestingly, Gconn, which reflects global integration of brain regions, does not show significant decreases in H, indicating the changes in BOLD scaling during ongoing headache may be more relevant to local neural functioning in this cohort.

This study had some limitations that should be acknowledged, as they qualify the interpretation of the results. First, the fMRI acquisition had a run length of ∼9 min, which is consistent with standard resting‐state protocols and balances the demands of adequate data and patient wakefulness (Birn et al., 2013; Tagliazucchi & Laufs, 2014). However, physiological time series on the order of 10^3^ or more are preferred for robust scaling analysis (Eke et al., 2000); future research employing multiple runs may be able to bypass these issues and explore scaling behavior over longer time scales. The sample sizes are also somewhat small for participant subgroups, although given the paucity of data examining PACS and post‐COVID headache, this remains a valuable dataset. In particular, the control group is relatively unique, having non‐COVID infection and similar post‐infection symptom profiles, compared to the more common literature practice of contrasting individuals with COVID‐19 against uninfected controls. The bootstrapped regression approach was chosen to preserve power in small and unbalanced samples, while the large effects sizes, robustness across scaling measures, and predefined hypotheses provide some internal validation of study findings. Nevertheless, it will be critical for future research to validate and replicate the current results. Future work should also collect more detailed information about headache symptoms, including phenotype (e.g., migraine vs. tension type), severity, and frequency, in order to gain a more nuanced understanding of the relationship between BOLD dynamics and headache. Finally, while the present study excluded participants with prior neurological conditions, future research should examine individuals with PACS and pre‐existing headache disorder. There is emerging evidence that the course of headache is significantly altered during COVID‐19 infection (Waliszewska‐Prosół & Budrewicz, 2021), and neuroimaging of these cohorts may provide greater insights into the mechanisms underlying post‐COVID headache.

Overall, the results of this study provide strong preliminary evidence that the headache component of PACS has a neural signature associated with power‐law scaling in resting‐state BOLD fMRI signals. This is congruent with existing literature where suppression of scaling behavior represents a more stressed, taxed state. These findings provide novel mechanistic insights into changes in brain dynamics associated with ongoing and recovered headache in individuals with PACS, potentially allowing the development of new tools for diagnosis and management of this emerging syndrome. Moreover, such insights into the mechanisms contributing to headache are relevant, given the importance of public education regarding headache management, for example, in terms of the functional changes that may lead to substance abuse headache (Katsuki et al., 2023).

## CONFLICT OF INTEREST STATEMENT

The authors report no disclosures relevant to the article.

### PEER REVIEW

The peer review history for this article is available at https://publons.com/publon/10.1002/brb3.3212.

## Supporting information

Appendix 1: MRI acquisition and preprocessing

Appendix 2: Comparison with other scaling estimators

Appendix 3: Sensitivity analysis of main study findings

Appendix 4: Self‐reported symptoms in the “other” category

## Data Availability

The datasets analyzed for this study can be found in the figshare data repository at: https://doi.org/10.6084/m9.figshare.22338370.
