# Supplementary material for: Persistent post‐COVID headache is associated with suppression of scale‐free functional brain dynamics in non‐hospitalized individuals
Source: Brain Behav. 2023 Oct 23;13(11):e3212. doi: 10.1002/brb3.3212 (PMC10636408; doi:10.1002/brb3.3212)
Supplement: Supplementary file 1 — Appendix 1: MRI acquisition and preprocessing [file BRB3-13-e3212-s004.docx]

**Appendix-1: MRI acquisition and preprocessing**

**MRI Acquisition:**

Participants were imaged at Sunnybrook Hospital using a Siemens Prisma MRI system operating at 3 Tesla (Siemens, Erlangen, Germany), with standard multi-channel head coil, using the previously published NeuroCOVID protocol ^1^. Structural imaging included 3D T1-weighted Magnetization Prepared Rapid Acquisition Gradient Echo (MPRAGE: inversion time (TI)/echo time (TE)/repetition time (TR) = 1100/4.37/2500 ms, flip angle (θ) = 7^o^, 192 sagittal slices with 240x240 mm field of view (FOV), 256x256 matrix, 1.0 mm slice thickness, 1.0x1.0 mm in-plane, 140 Hz/px bandwidth (BW)). Resting-state fMRI was acquired via 2D multi-slice T2*-weighted echo planar imaging (EPI: TE/TR =30/2130 ms, θ = 70^o^, 40 oblique-axial slices with 224x224 mm FOV, 64x64 matrix, 3.5 mm slice thickness, 3.5x3.5 mm in-plane, 2440 Hz/px BW), producing a series of 250 volumes (8:53 minutes in total). During acquisition, participants were instructed to lie still with their eyes open and not to focus on anything in particular.

**Data processing and quality control:**

Initial quality control: the DICOM headers were first checked to ensure consistent acquisition parameters, followed by manual inspection of T1-weighted anatomical and mean BOLD EPI images. The anatomical images were rated based on a system adapted and modified from Backhausen et al. ^2^. This system examined the scans on 7 criteria, each rated using a 4-point scale where 0=no/minimal artifact, 1=minor and localized artifact, 2=substantial and localized or minor and widespread, and 3=substantial and widespread. Overall quality ratings were based on the maximum score over the 7 items, with a value of 2 requiring additional post-processing quality checks and a value of 3 being grounds for rejection. Grading criteria included: brain coverage (the entire brain within the field of view), white-matter/grey-matter contrast (clear delineation of tissue boundaries), image sharpness (clearly defined edges of cortex, subcortex and cerebellum), ringing artifact, aliasing/ghosting artifact, zipper or spike artifact and susceptibility artifact. All anatomical images had overall ratings of 0 or 1 and were retained. The mean functional images were also rated using 6 of the 7 criteria applied to the anatomical data and using the same 4-point scale, with an overall rating based on the maximum score. The “ringing” criterion was excluded due to the challenges of a) identifying this feature in images with low spatial resolution, and b) the frequency of motion effects in the unprocessed data. All functional images had overall ratings of 0 or 1 and were retained.

Structural data processing: a hybrid pipeline was implemented, with initial clipping of excess neck via *3dAutoclip* to improve mask and warping quality and transforming the oblique scans to cardinal axes with *3dWarp*. Alignment to the *MNI152 2009* *SSW* template was then conducted with the analysis of functional neuroimages (AFNI; https://afni.nimh.nih.gov) *SSwarper* protocol and default settings but modified to use brain masks produced by the advanced normalization tools (ANTs; http://stnava.github.io/ANTs) *antsBrainExtraction* protocol, as it offered superior skull stripping performance based on visual inspection. The *SSwarper* protocol performed signal inhomogeneity correction, anisotropic smoothing and clipping at the 98^th^ percentile, followed by iterative nonlinear alignment to template with *3dQWarp*. The anatomical images were also segmented into grey matter (GM), white matter (WM) and cerebrospinal fluid (CSF) voxel maps using the functional MRI of the brain software library (FSL; https://fsl.fmrib.ox.ac.uk/fsl) *fast* protocol, with subsequent warping of tissue maps into template space via *3dNwarpApply*.

Functional data processing: the first 3 images in the timeseries were discarded from the run to allow for signal equilibration, based on inspection of the mean signal intensity curves. Outlier images were identified and replaced with more appropriate interpolated values using the *SPIKECOR* censoring algorithm ^3^ (code from: nitrc.org/projects/spikecor), and slice-timing correction was conducted using the AFNI *3dTshift* algorithm with default settings. Afterwards, the oblique scans were transformed to cardinal axes with *3dWarp*. Correction for within-run head movement was then performed by first identifying the minimum displacement volume: the volume was smoothed with a 6mm full-width at half maximum (FWHM) isotropic Gaussian kernel with *3dmerge* and masked with *3dAutomask*, then principal component analysis (PCA) was performed before identifying the medioid (the volume closest to the median PCA-space coordinates), as a robust non-outlier reference volume. The rigid-body alignment of each volume to the reference was obtained using *3dvolreg*, and the alignment of the reference to the anatomical image was obtained using *align_epi_anat.py*. The net transform of each functional volume into template space was then computed by concatenating motion correction, functional-to-anatomical, and anatomical-to-template warps using *cat_matvec* and *3dNWarpApply*, with images resampled to a final 3mm isotropic voxel resolution. Spatial smoothing was then done with a 6mm FWHM isotropic Gaussian kernel with *3dmerge*, and a participant brain mask was obtained using *3dAutomask*, with subsequent analyses undertaken using the intersection of the preprocessed functional images with the downsampled, masked anatomical images. Subsequent regression of nuisance covariates included detrending using a 2^nd^-order Legendre polynomial basis and regression of the first two PCs of the rigid-body motion parameter estimates (MPEs) obtained from the *3dvolreg* step. Physiological noise timeseries were also regressed out by obtaining masks of left and right lateral ventricles and cerebral white matter. For each of the three regions, PCA was used to identify the time-course explaining the most variance, which was then regressed from the data.

Post-processing checks and outlier detection: we visually inspected anatomical and functional brain masks overlaid on the images, along with aligned anatomical and mean functional volumes overlaid on the template. To control for the potential confounding effects of excess head movement, tests were also conducted to identify excessively high movement and associated effects in the functional imaging data. Head motion was summarized by calculating relative displacement (i.e., the root-mean-square difference of the 6 rigid-body MPEs, calculated between each volume and the preceding one), and total displacement (i.e., the root-mean-square difference of the 6 rigid-body MPEs, calculated between all possible volume pairs), based on MPEs obtained from the *3dvolreg* step. For each case, we measured both the mean and maximum over all calculated displacement values. We also calculated analogous metrics between BOLD images, to assess whether there were extreme changes in BOLD signal. A search was then conducted for any participants that were (a) significantly elevated in one of the four MPE metrics (based on a Gamma-fitted model; FDR=0.05), indicating excess movement; and (b) were similarly elevated in one of the four BOLD metrics, indicating that data processing failed to control for the motion effects on BOLD data. Using these criteria, we identified two (2) control participants that were significant outliers, who were then excluded from subsequent neuroimaging analyses.

**Checking for confounding effects of head motion**

Post-analysis checks also conducted to determine whether head motion amplitude was related to group membership, which may confound the group-level analyses of BOLD scaling. We focused on the four MPE metrics described in “Data processing and quality control” above, including the mean and maximum relative displacement and the mean and maximum total displacement. All checks were conducted on the log-transformed variables given the long distribution tails (i.e., all untransformed variables had kurtosis > 11.2; after transformation, all had kurtosis < 3.4). Regarding the main study effects of group differences in BOLD scaling, 2-sample bootstrap tests comparing all group pairs (control, COVID-H-, COVID-H+, COVID-Hr) found no significant differences in motion between groups, for any of the MPEs, after adjusting for multiple comparisons at an FDR of 0.05; uncorrected p-values showed a weak effect of lower motion in the COVID-H+ vs. control comparison (BSR=-1.91, p=0.048; maximum total displacement) with all other comparisons having |BSR|<1.70 and *p*>0.084. In addition, adjusting for head motion in the group-level regression model did not significantly alter the group coefficients of effect, for any of the MPEs (all |BSR|<1.28, *p*>0.149).

**REFERENCES**

1. MacIntosh BJ, Ji X, Chen JJ, et al. Brain structure and function in people recovering from COVID-19 after hospital discharge or self-isolation: a longitudinal observational study protocol. *Canadian Medical Association Open Access Journal.* 2021;9(4):e1114-e1119.

2. Backhausen LL, Herting MM, Buse J, Roessner V, Smolka MN, Vetter NC. Quality control of structural MRI images applied using FreeSurfer—a hands-on workflow to rate motion artifacts. *Frontiers in neuroscience.* 2016;10:558.

3. Churchill NW, Spring R, Afshin-Pour B, Dong F, Strother SC. An automated, adaptive framework for optimizing preprocessing pipelines in task-based functional MRI. *PloS one.* 2015;10(7):e0131520.
