# Supplementary material for: Persistent post‐COVID headache is associated with suppression of scale‐free functional brain dynamics in non‐hospitalized individuals
Source: Brain Behav. 2023 Oct 23;13(11):e3212. doi: 10.1002/brb3.3212 (PMC10636408; doi:10.1002/brb3.3212)
Supplement: Supplementary file 2 — Appendix 2: Comparison with other scaling estimators [file BRB3-13-e3212-s002.docx]

**Appendix-2: Comparison with other scaling estimators**

**Methods**

Among the many proposed estimators of scaling behaviour, significant tradeoffs in method performance may introduce bias into analyses and subsequent interpretation of results. To ensure that the PSD findings were not estimator-specific, the results were also compared against three other well-established scaling estimators, with distinct estimation procedures. This included detrended fluctuations analysis (DFA; a time-domain monofractal model) ^7,8^, wavelet monofractal analysis (WMA; a time-frequency monofractal model) ^9,10^ and wavelet leader multifractal analysis (WLM; a time-frequency multifractal method) ^11-13^. For the DFA method, scaling was calculated over window sizes ranging from 3 to 33 TR values (6.36 to 69.96 s). For the WMA and WLM methods, analysis was conducted with Daubechies wavelets with three vanishing moments calculated over an octave range of 2 to 4, which showed robust diagnostic performance. The WLM estimation was also performed over *q* values ranging from -10 to 10. Note that for the WLM approach, *H* was estimated via the first log-cumulant *c*_1_, which denotes the most frequently-occurring scaling parameter.

These collective measures were calculated for a 27-voxel cubic ROI placed on the CoM for each cluster identified as having significant group differences in scaling effects as outlined in Methods (“Regional effects of COVID-19 on BOLD scaling”), with subsequent averaging over all clusters to obtain a set of four model-specific *H* values per participant. Afterwards, bootstrapped GLM analyses were conducted to evaluate group differences for each of the BOLD measures, while adjusting for covariate effects of age and sex.

**Results**

For brain regions showing significant BOLD scaling effects of COVID-H+ relative to controls and COVID-H- (Fig. 2C, blue areas), comparisons between estimators within the peak regions indicates that the PSD estimator tends to obtain slightly higher *H* values than the others (0.062, [0.040, 0.082]), whereas the others do not differ significantly (Friedman test with post-hoc critical difference test, p=0.05). The PSD measures also exhibit relatively good concordance with DFA (0.78, [0.66, 0.87]), WMA (0.73, [0.58, 0.83]), WLMF (0.85, [0.76, 0.90]), with the wavelet multifractal approach showing best concordance.

Figure S1 depicts the average *H* exponent values for the different estimators, plotted for significant regions identified in Fig. 2C. It can be seen that the effects generalize across estimators, with significantly reduced *H* values in COVID-H+ relative to both controls and COVID-H- groups seen for PSD in Fig. S1A (-0.245, [-0.322, -0.170], BSR=-6.31) and similarly highly reliable effects, albeit with diminished absolute and relative effect sizes, for DFA in Fig. S1B (-0.100, [-0.157, -0.037], BSR=-3.28), for WMA in Fig. S1C (-0.186, [-0.266, -0.103], BSR=-4.46) and for WLMF in Fig. S1D (-0.196, [-0.273, -0.116], BSR=-4.90), all with p<0.001. These results indicate that the observed effects within consensus brain regions do not depend substantially on the choice of scaling estimator.


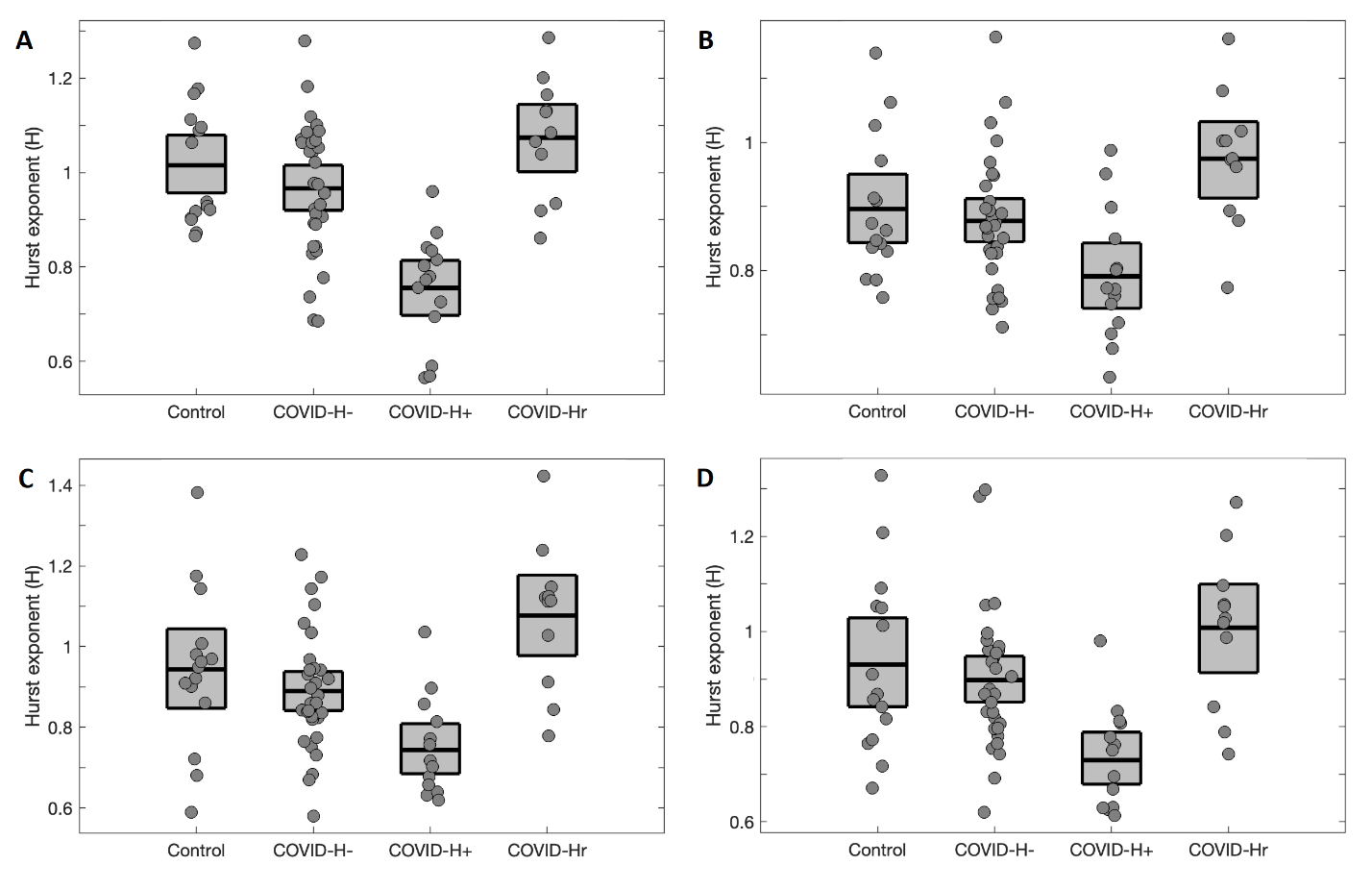


**Figure S1**: effects of COVID-19 and post-COVID headache on different estimators of BOLD scaling behaviour, for brain regions showing significant effects of ongoing headache (Fig. 2C, blue areas). Results are plotted for controls and COVID-19 groups without headache (COVID-H-) with ongoing headache (COVID-H+) and resolved headache (COVID-Hr). Results are shown for (A) power spectral density (PSD) approach, (B) detrended fluctuations analysis (DFA) approach, (C) wavelet monofractal analysis (WMA) approach and (D) wavelet leader multifractal (WLMF) approach. Boxes denoted group means and bootstrapped 95%CIs of the mean per group.

For brain regions showing significant BOLD scaling effects of Hr relative to controls and H- (Fig. 2C, red areas), comparison between estimators within the peak regions indicates that the PSD estimator tends to obtain slightly higher *H* values than the others (0.082, [0.051, 0.110]), whereas the others do not differ significantly (Friedman test with post-hoc critical difference test, p=0.05). The PSD measures also exhibit relatively good concordance with DFA (0.84, [0.75, 0.90]), WMA (0.73, [0.60, 0.82]), WLMF (0.88, [0.81, 0.93]), with the wavelet multifractal approach showing best concordance.

Figure S2 depicts the average *H* exponent values for the different estimators, plotted for significant regions identified in Fig. 2C. It can be seen that the effects generalize across estimators, with increased *H* values in COVID-Hr relative to both control and COVID-H- groups, seen for PSD in Fig. S2A (0.264, [0.162, 0.370], BSR=4.88, p<0.001) and similarly highly reliable effects, with comparable absolute but diminished relative effect sizes, for DFA in Fig. S2B (0.179, [0.102, 0.254], BSR=4.59), for WMA in Fig. S2C (0.275, [125, 0.433], BSR=3.60) and for WLMF in Fig. S2D (0.270, [0.145, 0.406], BSR=3.98), all with p<0.001. These results indicate that the observed effects within consensus brain regions do not depend substantially on the choice of scaling estimator.


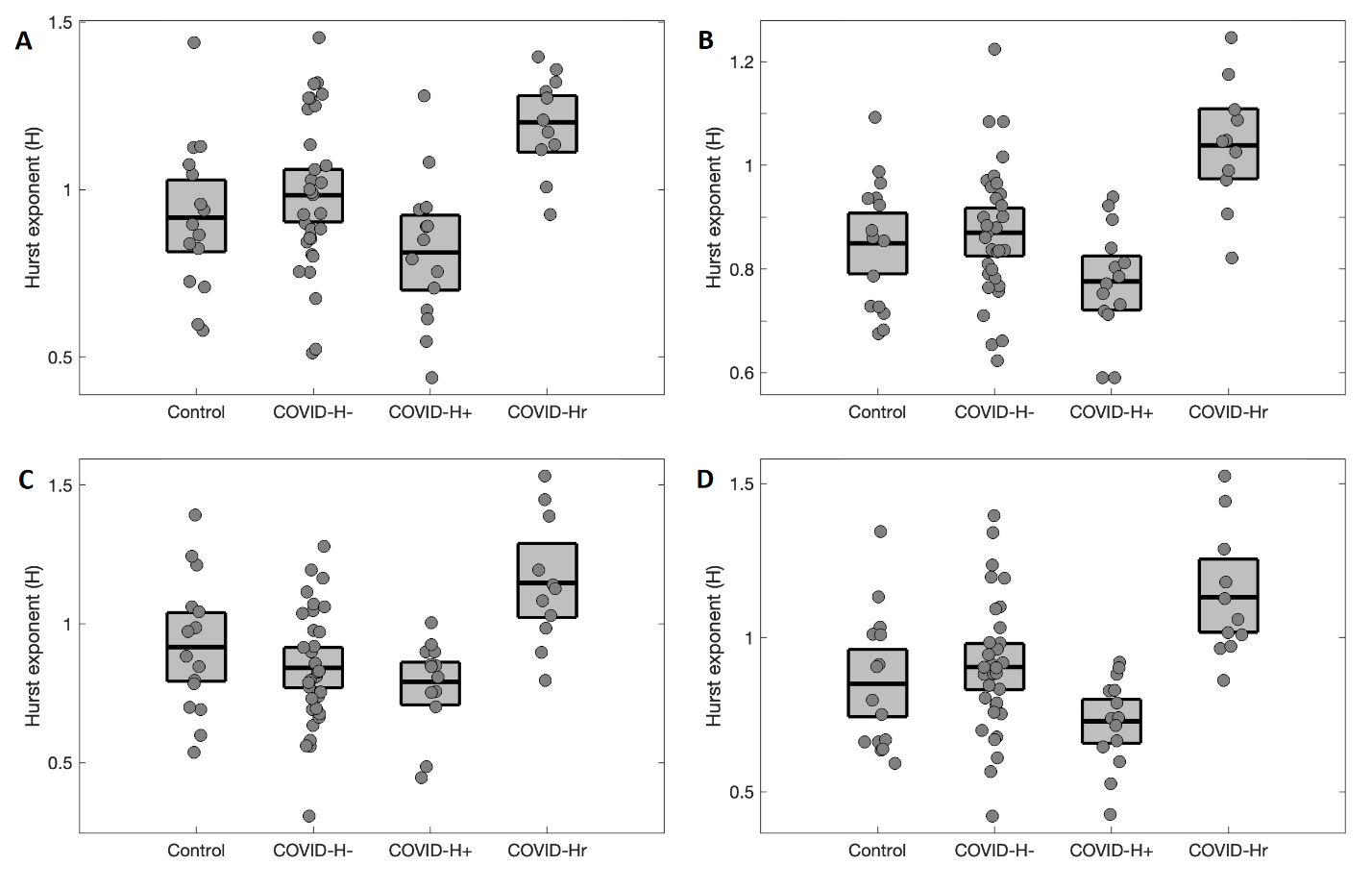


**Figure S2**: effects of COVID-19 and post-COVID headache on different estimators of BOLD scaling behaviour, for brain regions showing significant effects of resolved headache (Fig. 2C, red areas). Results are plotted for controls and COVID-19 groups without headache (COVID-H-) with ongoing headache (COVID-H+) and resolved headache (COVID-Hr). Results are shown for (A) power spectral density (PSD) approach, (B) detrended fluctuations analysis (DFA) approach, (C) wavelet monofractal analysis (WMA) approach and (D) wavelet leader multifractal (WLMF) approach. Boxes denoted group means and bootstrapped 95%CIs of the mean per group.
