# Supplementary material for: Persistent post‐COVID headache is associated with suppression of scale‐free functional brain dynamics in non‐hospitalized individuals
Source: Brain Behav. 2023 Oct 23;13(11):e3212. doi: 10.1002/brb3.3212 (PMC10636408; doi:10.1002/brb3.3212)
Supplement: Supplementary file 3 — Appendix 3: Sensitivity analysis of main study findings [file BRB3-13-e3212-s001.docx]

**Appendix-3: Sensitivity analysis of main study findings**

**Methods**

The robustness of the study findings was evaluated with respect to regression model specification. In the main study analyses, a general linear model (GLM) was fitted on the global Hurst exponent (*H*_glob_) data from all participant subgroups (control, COVID-H-, COVID-H+, COVID-Hr), with binary regressors denoting membership in the different COVID-19 subgroups; the GLM also adjusted for effects of age and sex. The latter were based on prior literature showing significant effects of these variables on *H* (Churchill, Hutchison, Graham, & Schweizer, 2020; Churchill et al., 2016). The sensitivity analyses in this section evaluate (1) the overall importance of individual regressors in the model, and (2) the impact of including/excluding model covariates on the main regressors of interest (i.e., the COVID-19 subgroup effects).

For sensitivity analysis, we considered both the “original” set of 5 regressors (3 subgroup labels, age, sex), and an “expanded” set of regressors that includes 4 other potential covariates of interest: years of education (related to cognitive reserve), days from symptom onset to MRI (reflecting time-evolving pathophysiology), vaccination status (potentially affecting the severity of COVID-19 infection), and probable COVID-19 variant (potentially affecting disease manifestation). Days from symptom onset to MRI was log-transformed due to long distribution tails (skew: 1.3, kurtosis: 5.2) and vaccination status was coded as a binary variable (1 = any vaccine prior to PCR test date / 0 = no vaccine) due to its relatively low prevalence in the groups (11%). Probable COVID-19 variant was also coded as a binary variable (1=probable wild-type / 0=probable later variant), based on the dominant strain in Ontario at the time of the PCR testing (Ontario COVID-19 Science Advisory Table, 2022); the majority of individuals (78%) were considered to have a probable wild-type infection, with their PCR tests occurring prior to March 4, 2021 when the wild-type strain fell below 50% of cases in Ontario.

For the first aim, we examined all possible combinations of regressors included in the GLM and measured the overall difference in explanatory power for models with/without each regressor of interest. For the “original” regressor set there is a total of 32 possible models (including an intercept-only model), while for the “expanded” regressor set there is a total of 512 possible models. Evidence for each model was assessed via Akaike information criterion with small-sample correction (AICC) (Anderson & Burnham, 2004). For linear regression with *K* parameters, sample size *N* and model likelihood *L*, this is:

$$AICC=-2log(L)+2K+2K(K+1)/(N-K-1)$$

In model comparison, the optimal model minimizes AICC, which balances model fit to the data (i.e., high values of *L*) against model parsimony (i.e., low values of *K*). For the *i*^th^ model, performance is assessed in terms of difference from the optimal model, ${\Delta AICC}_{i}={AICC}_{i}-{AICC}_{min}$ and the relative model likelihood calculated as $L_{AICC,i}=e^{-{\Delta AICC}_{i}/2}$, which is then normalized to obtain weights $w_{AICC,i}=L_{AICC,i}/\sum_{j} L_{AICC,j}$. We can get the Akaike inclusion probability (AIP) for a given variable by summing over all $w_{AICC,i}$ that contain the variable of interest. This value can be considered the probability that the best approximating model contains the variable of interest. The set of AIP weights were calculated for all variables, in both the “original” and “expanded” models.

For the second aim, we held the COVID-19 subgroup regressors fixed and examined all possible combinations of the remaining covariates included in the GLM, by measuring the variability of coefficient values for the COVID-19 subgroup regressors across the different models. For the “original” regressor set there is a total of 4 possible models and for the “expanded” regressor set there are 64 possible models. For each COVID-19 subgroup regressor, the overall influence of covariate choices on the regression coefficient was assessed by taking the standard deviation of coefficient values over all model combinations, divided by the mean coefficient value over all model combinations, multiplied by 100 (i.e., the percent coefficient of variation). The specific impact of each covariate on the COVID-19 subgroup regressor’s coefficient was also assessed, by measuring the mean percent change in coefficient value between models that included/excluded the covariate of interest. The results were also compared to a “null” regressor that had no specific explanatory power or association with other covariates, generated as a random Gaussian signal. Effects for the null regressor were assessed over 2000 simulation runs, with 95% confidence intervals (95%CIs) drawn for comparison purposes.

**Results**

For the first aim, Table S1 below summarizes the importance of each variable in the GLM in terms of their AIP value. In both the original and expanded models, the regressor for COVID-19 without headache (H-) has a relatively weak inclusion probability, while COVID-19 with headache (H+) has a strong inclusion probability and COVID-19 recovered from headache (Hr) has an intermediate AIP value. These results are consistent with the relative strength of effects seen in the main study analyses. Among the other covariates of interest, age appears to be most relevant, with an intermediate inclusion probability; all other covariates have relatively low AIP values, indicating low probability of being included in the optimal model.

For the second aim, overall variability in the effects of COVID-19 subgroup due to variable choice in the original model was low to moderately-low for COVID-19 without headache (11.60%), COVID-19 with headache (5.50%) and COVID-19 recovered from headache (7.85%). In the expanded model, variability of effects was somewhat increased for COVID-19 without headache (20.99%) but less so for COVID-19 with headache (6.16%) and COVID-19 recovered from headache (12.83%). Table S2 below summarizes the average impact of each covariate on the COVID-19 subgroup coefficients. Effects of individual covariates were generally modest, with greatest influence seen for age on COVID-19 with headache and COVID-19 recovered from headache, in both original and expanded models. However, none of the regressors showed an impact exceeding the 95%CI of the “null” model, hence we conclude that study findings are generally robust to covariate choice.

**Table S1**: Akaike Inclusion Probabilities (AIP) for each variable, indicating likelihood of their inclusion in the optimal GLM. Variables include COVID-19 without headache (H-), COVID-19 with ongoing headache (H+), COVID-19 that has recovered from headache (Hr), age, sex, years of education (educ.), days from symptom onset to MRI (days), vaccine status (vacc.), and probable viral variant (v. var.).

|  | H- | H+ | Hr | age | Sex | educ. | days | vacc. | v. var. |
| --- | --- | --- | --- | --- | --- | --- | --- | --- | --- |
| Original | 0.30 | 0.95 | 0.65 | 0.62 | 0.24 | -- | -- | -- | -- |
| Expanded | 0.29 | 0.95 | 0.57 | 0.61 | 0.23 | 0.24 | 0.26 | 0.39 | 0.24 |

**Table S2**: overall effect of including each covariate in the GLM, in terms of the impact on subgroup coefficients for COVID-19 without headache (H-), COVID-19 with ongoing headache (H+), COVID-19 that has recovered from headache (Hr). Effects are evaluated in terms of the mean percent change in subgroup coefficient value, for all models that include the covariate of interest, compared to all models that exclude the covariate of interest. Covariates of interest include age, sex, years of education (educ.), days from symptom onset to MRI (days), vaccine status (vacc.), and probable viral variant (v. var.). For reference, the effects of a “null” regressor (random Gaussian noise) are also included, with the 95% confidence interval (95%CI) across 2000 simulation runs.

|  |  | age | sex | educ. | days | vacc. | v. var. | null, 95%CI |
| --- | --- | --- | --- | --- | --- | --- | --- | --- |
| Original | H-  H+  Hr | -0.53%  3.19%  2.78% | -0.09%  -0.25%  0.07% | --  --  -- | --  --  -- | --  --  -- | --  --  -- | [-3.65%, 4.04%]  [-3.75%, 3.92%]  [-3.29%, 3.45%] |
| Expanded | H-  H+  Hr | -0.41%  3.55%  -2.66% | -0.02%  -0.03%  -0.04% | -0.18%  0.11%  -0.30% | -1.28%  -2.00%  -1.14% | 0.09%  0.69%  -3.46% | 0.05%  0.24%  -0.09% | [-4.22%, 4.11%]  [-3.92%, 3.66%]  [-3.85%, 3.69%] |

**REFERENCES**

Anderson, D., & Burnham, K. (2004). Model selection and multi-model inference. *Second. NY: Springer-Verlag, 63*(2020), 10.

Churchill, N. W., Hutchison, M. G., Graham, S. J., & Schweizer, T. A. (2020). Scale‐free functional brain dynamics during recovery from sport‐related concussion. *Human brain mapping, 41*(10), 2567-2582.

Churchill, N. W., Spring, R., Grady, C., Cimprich, B., Askren, M. K., Reuter-Lorenz, P. A., . . . Berman, M. G. (2016). The suppression of scale-free fMRI brain dynamics across three different sources of effort: aging, task novelty and task difficulty. *Scientific reports, 6*, 30895.

Ontario COVID-19 Science Advisory Table. (2022). Ontario Dashboard: Percentage of Cases Caused by Different Variants in Ontario. Retrieved from
