# Supplementary material for: Persistent post‐COVID headache is associated with suppression of scale‐free functional brain dynamics in non‐hospitalized individuals
Source: Brain Behav. 2023 Oct 23;13(11):e3212. doi: 10.1002/brb3.3212 (PMC10636408; doi:10.1002/brb3.3212)
Supplement: Supplementary file 4 — Appendix 4: Self‐reported symptoms in the “other” category [file BRB3-13-e3212-s003.docx]

**Appendix-4: self-reported symptoms in the “other” category**

**Methods**

Along with pre-specified symptoms (fever, cough, sore throat, shortness of breath, fatigue, gastrointestinal issues, problems with smell/taste, headache), participants were able to fill out up to 10 additional “other” symptoms that they had experienced concurrent with, or subsequent to, the study-relevant viral infection and PCR test. Symptoms were again rated as either ongoing or resolved. These symptoms were collected and presented in tabular format, for controls and for individuals with COVID-19 without headache symptoms (COVID-H-), with ongoing symptoms (COVID-H+) and with recovered symptoms (COVID-Hr).

**Results**

Table S1 below shows substantial inter-individual heterogeneity of reporting within the “other” category, for both the control and COVID-19 subgroups. In terms of ongoing symptoms qualitative assessment suggests that cognitive issues are more prevalent in the COVID-H+ (4/14 (29%)) and COVID-Hr (2/11 (18%)) groups, compared to the control (0/17) and COVID-H- (1/32 (3%)) groups. Pain issues are also more common in the COVID-H+ (4/14 (29%)) group than in the control (2/17 (12%)), COVID-H- (2/32 (6%)) or COVID-Hr (1/11 (9%)) groups. Interestingly, signs of dysautonomia (high blood pressure, elevated heart rate, heart palpitations, exertional malaise) appear slightly more prevalent in controls (3/17 (17%)) and COVID-Hr (2/11 (18%)) groups, compared to COVID-H- (3/32 (9%)), COVID-H+ (1/14 (7%)) groups.

**Table S1**: list of symptoms reported in the “other” category for each group, by participant. This included controls and individuals with COVID-19 without headache symptoms (COVID-H-), with ongoing symptoms (COVID-H+) and with recovered symptoms (COVID-Hr). Ongoing symptoms are shaded in dark grey, recovered symptoms are shaded in light grey.

|  |  | **symptom-1** | **symptom-2** | **symptom-3** | **symptmo-4** | **symptom-5** | **symptom-6** |
| --- | --- | --- | --- | --- | --- | --- | --- |
| control | 1 | swollen lymph nodes | light headed | heart palpitations | burning lungs |  |  |
|  | 2 | chest pain/pressure | muscle ache | arm pain/weakness |  |  |  |
|  | 3 | body pain | face numbness/pain | tingling |  |  |  |
|  | 4 | elevated heart rate | head pressure | arm/back pain | chest pain |  |  |
|  | 5 | elevated heart rate | chest congestion |  |  |  |  |
|  | 6 | sinus congestion | post-nasal drip |  |  |  |  |
| COVID-H- | 1 | cognitive issues | high blood pressure | elevated heart rate | chest pain | hair loss | rash |
|  | 2 | hearing issues | tingling | hand tremor | eye fatigue | foot numbness |  |
|  | 3 | dizziness | chest pressure |  |  |  |  |
|  | 4 | high blood pressure |  |  |  |  |  |
|  | 5 | high blood pressure |  |  |  |  |  |
|  | 6 | post-nasal drip |  |  |  |  |  |
|  | 7 | chest pain |  |  |  |  |  |
|  | 8 | body ache |  |  |  |  |  |
|  | 9 | migraine (non-headache) |  |  |  |  |  |
|  | 10 | chills | cold sweat | back ache |  |  |  |
|  | 11 | chills | light sensitivity |  |  |  |  |
|  | 12 | rash | elevated heart rate |  |  |  |  |
|  | 13 | vomitting | vision issues |  |  |  |  |
|  | 14 | chest pain |  |  |  |  |  |
|  | 15 | thyroid swelling |  |  |  |  |  |
|  | 16 | cognitive issues |  |  |  |  |  |
|  | 17 | cognitive issues |  |  |  |  |  |
|  | 18 | body ache |  |  |  |  |  |
|  | 19 | sore muscles |  |  |  |  |  |
|  | 20 | nausea |  |  |  |  |  |
|  | 21 | muscle spasm |  |  |  |  |  |
|  | 22 | msucle ache/pain |  |  |  |  |  |
| COVID-H+ | 1 | cognitive issues | dizziness | joint pain |  |  |  |
|  | 2 | dizziness | vertigo | exertional malaise |  |  |  |
|  | 3 | cognitive issues | swollen brain feeling | swelling in extremities |  |  |  |
|  | 4 | chest tightness | ear pain | spleen pain |  |  |  |
|  | 5 | cognitive issues | side pain |  |  |  |  |
|  | 6 | sinus pain | ear pain |  |  |  |  |
|  | 7 | migraine | sinus congestion |  |  |  |  |
|  | 8 | cognitive issues |  |  |  |  |  |
|  | 9 | chills | phlegm | body ache | chest clearing |  |  |
|  | 10 | chest pain | back pain |  |  |  |  |
|  | 11 | chest pain |  |  |  |  |  |
| COVID-Hr | 1 | neck and chest pain | bone pain | muscle pain | elevated heart rate | foot itch |  |
|  | 2 | heart palpitations | muscle spasm (ear) | sinus pressure/pain | light sensitivity |  |  |
|  | 3 | vertigo | cognitive issues | face tingling |  |  |  |
|  | 4 | cognitive issues | light sensitivity | bronchitis |  |  |  |
|  | 5 | runny nose | sneezing | post-nasal drip | cognitive issues |  |  |
|  | 6 | cold sweat |  |  |  |  |  |
|  | 7 | body ache |  |  |  |  |  |
|  | 8 | cognitive issues |  |  |  |  |  |
|  | 9 | nerve pain (back/ankles) |  |  |  |  |  |
